# Strangulated Inguinal Hernia With Intraperitoneal Proximal Bowel Perforation: An Unusual Presentation

**DOI:** 10.7759/cureus.84735

**Published:** 2025-05-24

**Authors:** Chaitanya P Garg, Qahtan A Al Dulaimi

**Affiliations:** 1 Surgery, Saqr Hospital, Emirates Health Services, Ras Al Khaimah, ARE; 2 Surgery, RAK Medical & Health Sciences University, Ras Al Khaimah, ARE

**Keywords:** darn repair, inguinal hernia, intestinal obstruction, intestinal perforation, surgical case report

## Abstract

Strangulated inguinal hernias are a common surgical emergency that requires prompt intervention to save the bowel. Perforation of the strangulated bowel loop inside the inguinal canal due to delayed presentation is known, but the occurrence of intraperitoneal proximal bowel perforation is exceedingly rare. This poses a unique challenge in terms of the surgical incision required and the type of hernia repair to be done. This case report describes a 44-year-old male who presented with a strangulated right inguinal hernia along with peritonitis. A midline exploratory laparotomy revealed two perforations in the proximal ileum, which were sutured primarily. The entire bowel was checked for any obvious gross pathology. The bowel wall did not appear inflamed, thickened, or constricted at any point. The strangulated bowel in the inguinal canal was viable and was reduced successfully, and the hernia was repaired through a separate inguinal incision using the nylon Darning technique. The patient had a full recovery with no recurrence at six-month follow-up.

## Introduction

Inguinal hernias occur when abdominal contents protrude through the inguinal canal. They usually present as a bulge in the groin that becomes prominent with intraabdominal straining and reduces when lying down. Sometimes, the contents get stuck inside the inguinal canal, leading to obstruction. Prolonged obstruction can lead to bowel strangulation due to compromised blood supply in approximately 2-10% of instances [1]. If not addressed quickly, this can result in ischemia, necrosis, and bowel perforation. These perforations typically occur in the constricted section of the bowel loop within the hernial sac located in the inguinal canal. However, a perforation of the proximal small intestine inside the abdominal cavity along with intestinal obstruction is very uncommon and presents a considerable therapeutic difficulty. These situations necessitate an exploratory laparotomy to address the bowel perforation with peritonitis, along with a search to find the cause of the perforation.

In elective settings, a mesh repair of inguinal hernias is the standard of care. However, in contaminated hernias, there is a dilemma of employing mesh to fix the hernia, given the elevated risk of mesh infection in the presence of peritonitis [2]. In such cases, the Darning technique of hernia repair can be used, or deferred interval elective mesh repair of inguinal hernia can be planned. This case report describes an unusual occurrence of a strangulated right indirect inguinal hernia alongside an intraperitoneal proximal ileal perforation, a rare yet significant surgical emergency.

## Case presentation

**Figure 1:**
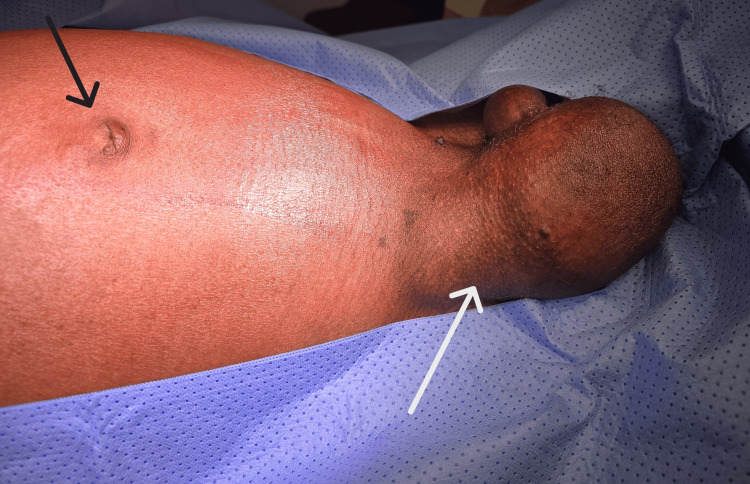
Preoperative image of the patient The image is showing distended abdomen, central umbilicus (black arrow) and right inguinoscrotal swelling (white arrow).

A 44-year-old male presented to the emergency department with acute abdominal pain, distention, vomiting, and constipation for one day. He had no fever. The patient reported a long-standing history of a reducible right inguinoscrotal hernia that had become irreducible and painful over the past 24 hours. The patient did not undergo elective surgical repair of his hernia earlier, while it was still reducible, due to financial constraints. On physical examination, the patient was afebrile and in distress. He had a distended abdomen, with marked generalized tenderness, and a palpable, tender, irreducible swelling in the right inguinoscrotal region (Figure 1).

**Figure 2:**
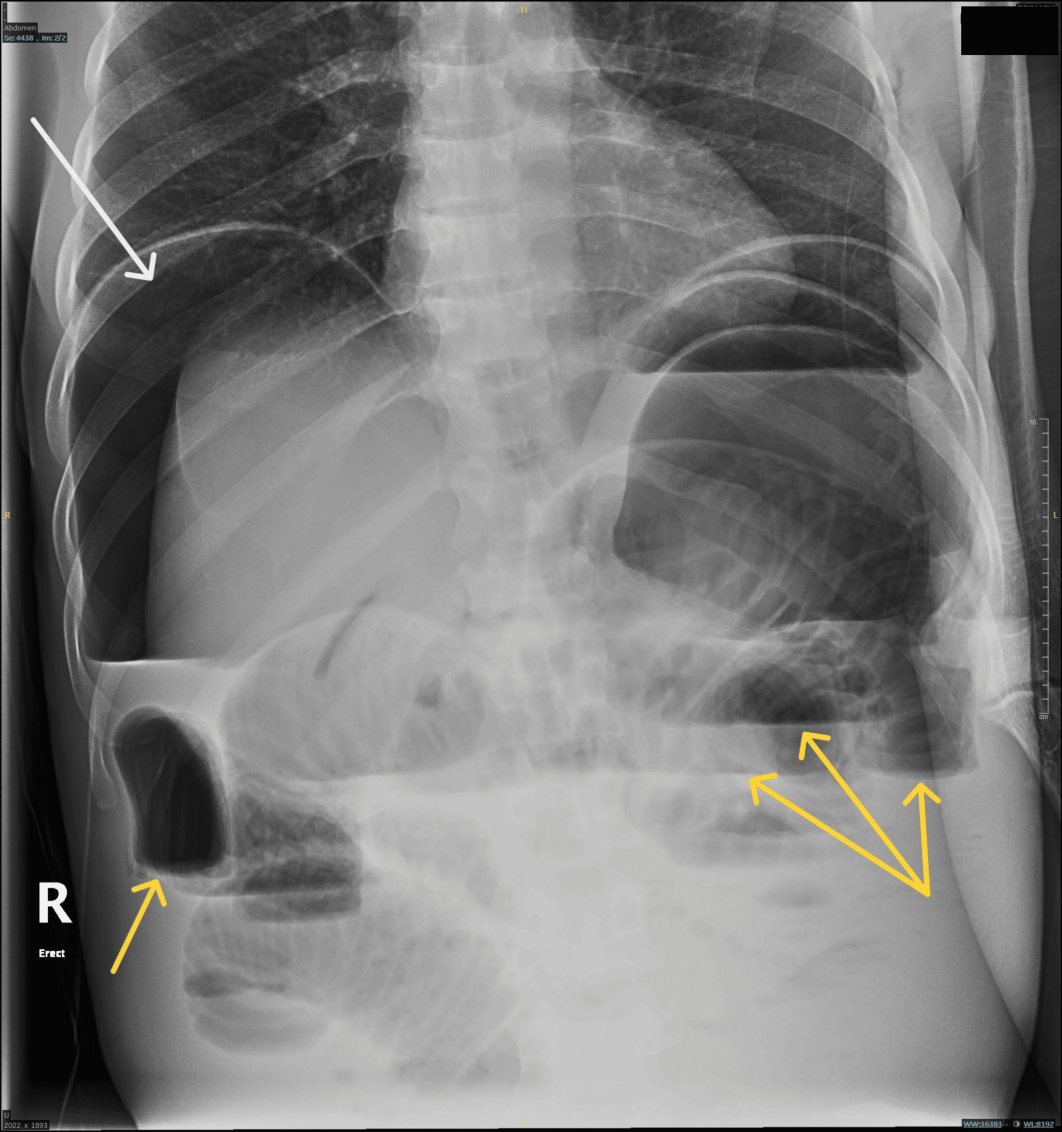
Plain X-ray abdomen The image is showing pneumoperitoneum (white arrow) and multiple air fluid levels (yellow arrows).

Intravenous fluids were started; laboratory and radiological tests were ordered. Laboratory investigations revealed leucocytosis, elevated C-reactive protein and normal electrolytes. Plain abdominal radiographs showed free air under the diaphragm and multiple air-fluid levels suggestive of hollow viscus perforation with intestinal obstruction (Figure 2).

**Figure 3:**
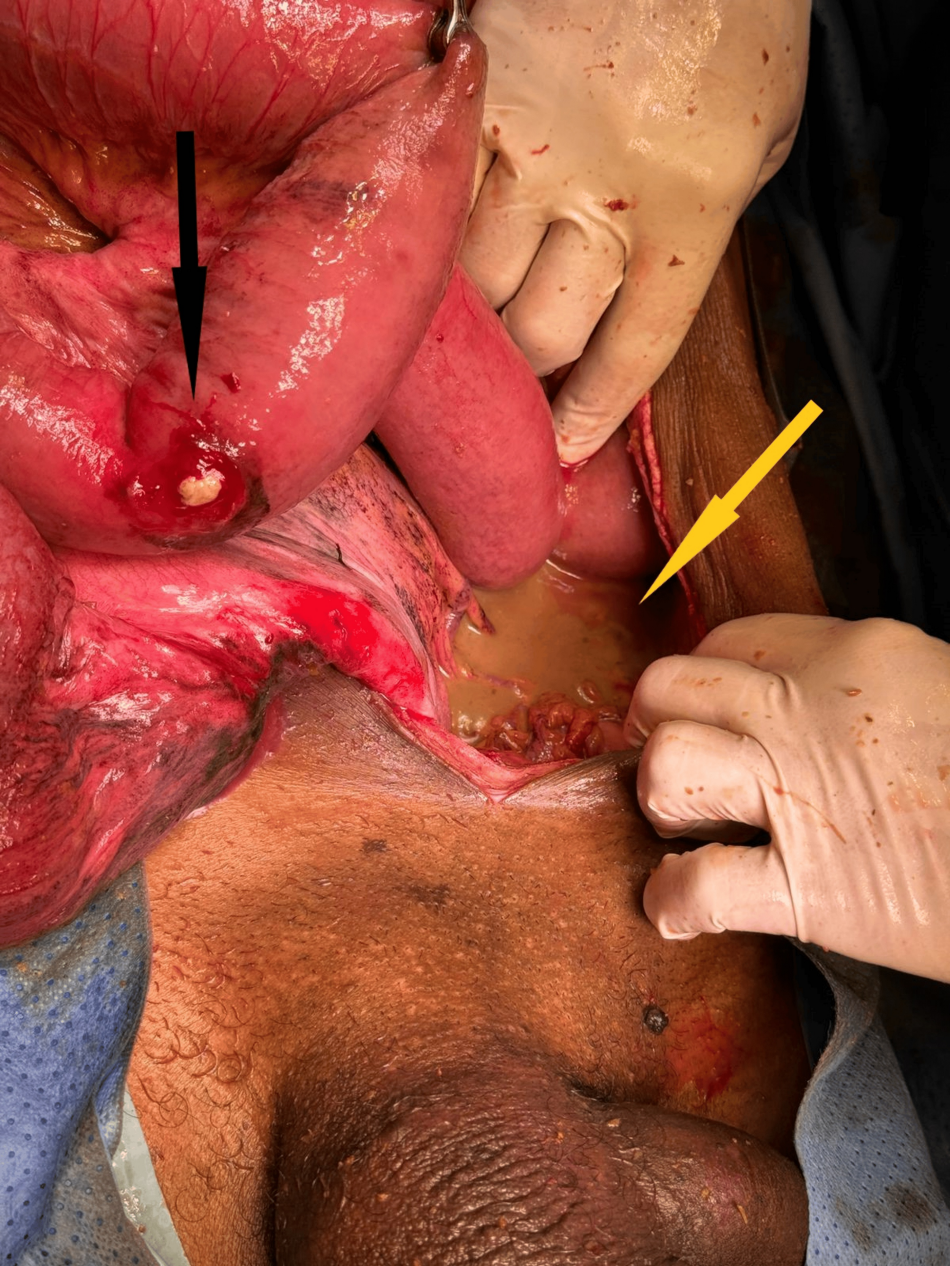
Intraoperative image The image is showing ileal perforation (black arrow) and purulent fluid (yellow arrow) in abdomen.

Given the severity of the presentation, an emergency surgery was performed. A midline exploratory laparotomy revealed extensive purulent fluid throughout the peritoneal cavity, indicating intraperitoneal sepsis. Two small perforations were found in the proximal ileum, about 130 cms and 140 cms proximal to the ileocecal junction, with no obvious cause of the perforations (Figure 3).

The entire bowel was checked for any obvious pathology. The bowel wall did not appear inflamed, thickened, or constricted at any point. The obstructed bowel in the right inguinal canal was located about 50 cms proximal to the ileocecal junction. It was gently pulled inside the abdominal cavity along with dark brownish fluid. The bowel had thickened walls but appeared viable. It was wrapped in abdominal gauze soaked with warm saline for a few minutes, and peristalsis was observed in the bowel to confirm its viability.

The edges of the perforation were not indurated or inflamed and were sutured primarily using interrupted absorbable sutures, without taking a biopsy. A thorough peritoneal lavage was performed with warm saline. No bowel resection was required as the strangulated bowel appeared viable after reduction. Two intraabdominal drains were placed, and the abdomen was closed in layers. Given the fact that the patient had ignored repair of his hernia earlier due to financial reasons, a decision to repair the hernia in the same sitting was taken. A separate incision was made in the right inguinal region, the indirect hernia sac was dissected, ligated and excised, and the posterior wall was repaired using the nylon Darning technique. A prosthetic mesh repair was not done due to the presence of concomitant peritonitis.

**Figure 4:**
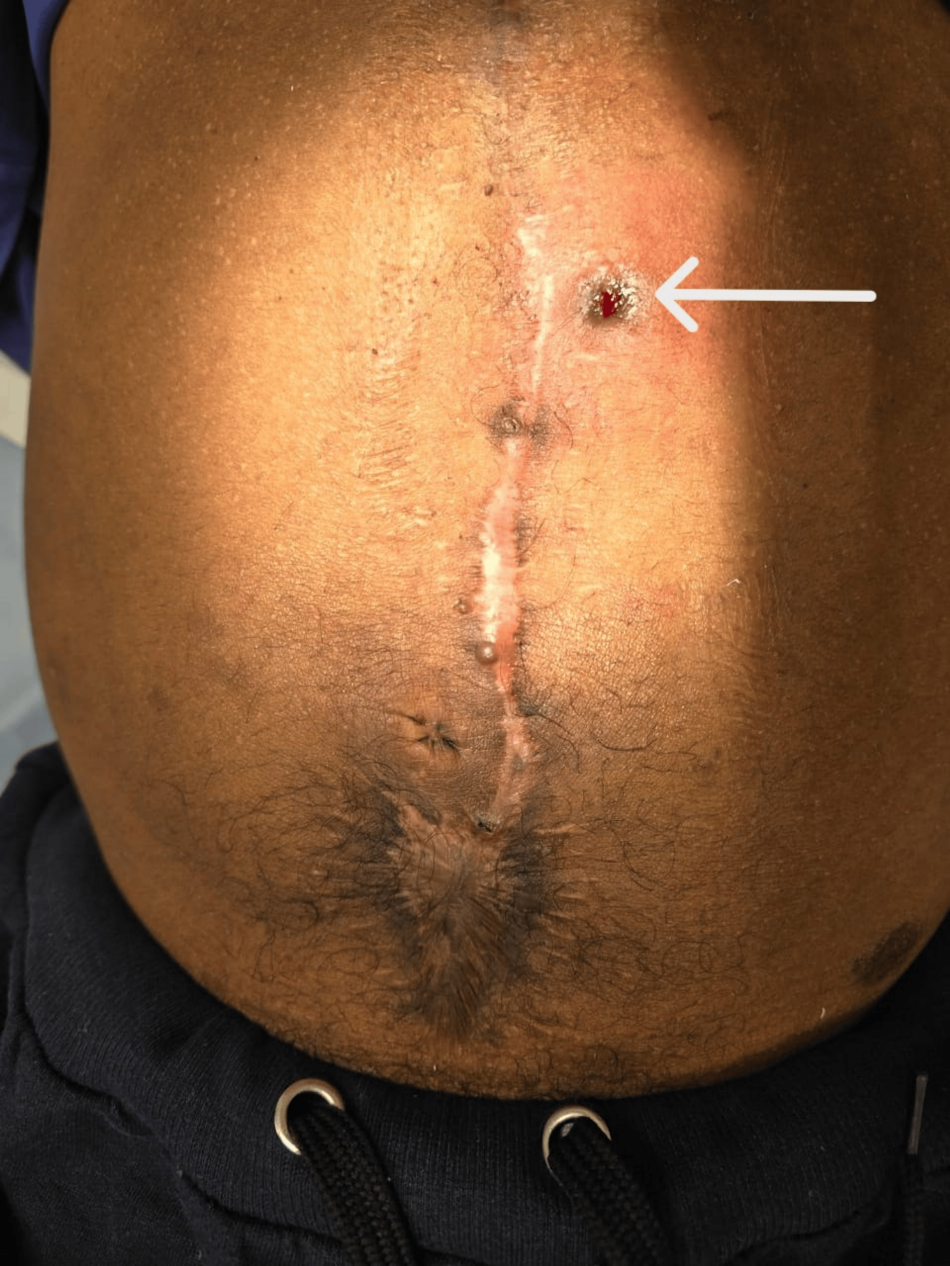
Postoperative patient image (after six months) The image is showing healed abdomen incision at six-month follow up with a healing stitch sinus on left side (white arrow).

The patient was managed postoperatively with intravenous antibiotics and supportive care. He showed steady improvement, and bowel function returned by the third postoperative day. The patient developed abdominal midline wound dehiscence on the fourth postoperative day and underwent secondary suturing of the abdominal wall defect. The inguinal incision healed primarily without any complications. The patient was discharged on the seventh postoperative day with no other complications and remained asymptomatic at the six-week, 12-week and six-month follow-up (Figure 4), with complete healing of the wound.

## Discussion

Groin hernias are among the most common problems encountered by general surgeons worldwide and may have significant morbidity when they present with incarceration or strangulation [3]. This case report presents an unusual presentation of inguinal hernia that required laparotomy for the perforation and an alternative hernia repair technique instead of the standard mesh repair.

It is well known that the chances of incarceration are higher in indirect inguinal hernias than in direct hernias due to the longer path it follows into the scrotum, encountering two potential constriction points: the deep and superficial inguinal rings. Hence, patients with indirect inguinal hernias are recommended to undergo surgical repair at the earliest, while the hernia is still reducible. Our patient had a reducible indirect inguinal hernia for a long time but did not undergo elective surgical repair due to financial constraints.

Once a hernia gets incarcerated, there is a risk of obstruction and strangulation. They often require emergency exploration, with 15-20% of cases requiring bowel resection, thereby increasing the complications and the hospital stay [4,5]. Typically, bowel perforation in the setting of a strangulated hernia would be expected to occur at the site of strangulation or distal to it due to the compromised blood supply. Such perforations would be confined to the hernial sac in the groin. The proximal intraperitoneal bowel progressively dilates due to obstruction but doesn’t perforate. This case is extremely rare due to the presence of two perforations in the proximal ileum without any gangrenous changes in the bowel.

Diagnosing intraperitoneal bowel perforation is crucial, so that a midline laparotomy can be performed first, allowing control of infection and bowel repair. Then the hernia can be repaired using a separate inguinal incision. This minimizes inguinal wound contamination and the possibility of surgical site infection. There were no complications at the inguinal hernia site repair in our case.

There are studies advocating diagnostic laparoscopy in small bowel obstruction in selective cases [6,7]. Most of the reported cases are due to adhesions and obstructed hernia, but the excluded cases are those with peritonitis. They also reported a higher rate of conversion to open surgery in cases with grossly dilated bowel, due to the technical difficulty of performing laparoscopy along with a risk of iatrogenic bowel injury. Our case had a distended abdomen due to dilated bowel and peritonitis due to perforation; hence, an open surgical approach was chosen.

Bakhteyar et al. reported a case of strangulated direct hernia with intraperitoneal jejunal necrosis and perforation [8]. They postulated that the jejunal necrosis occurred due to traction on the mesentery from the incarcerated hernia, resulting in compromise of the jejunal blood supply [8]. The presence of proximal perforation suggests an atypical pathophysiological process, possibly involving delayed presentation, excessive intraluminal pressure, or a pre-existing condition, weakening the bowel wall. Different etiologies have been reported for spontaneous small bowel perforation, including Crohn’s disease, celiac disease, and neoplastic disorders [9]. No obvious cause of the perforations could be identified in our case. The edges of the perforation were not indurated or inflamed and were sutured primarily without taking a biopsy. A literature search on PubMed, Scopus, and Google Scholar did not reveal any reported cases similar to our case.

Prosthetic mesh repair in incarcerated or strangulated groin hernia is controversial, especially when bowel resection is needed. There is a higher risk of mesh infection in such situations, which can significantly increase the morbidity. A study reported a 10-year experience with the use of prosthetic mesh repair in the management of acutely incarcerated and/or strangulated groin hernias. It concluded that the presence of non-viable intestine cannot be regarded as a contraindication for prosthetic repair [10]. However, this study did not specify using mesh in the presence of contamination with pus. On the contrary, a recent meta-analysis comprising 12,402 patients concluded that in the setting of bowel resection, mesh repair might increase the incidence of surgical site infection [11]. To avoid such risk, some centers advocate a delayed elective repair of hernia in such situations. However, considering the fact that our patient had ignored his hernia earlier, it was decided that we should repair his hernia in the same sitting.

The Desarda technique of pure tissue repair and the Darning technique using nylon suture material for hernia have been advocated for emergency hernia repairs [12,13]. We used the Darning technique as we were more familiar with it. A Darn inguinal hernia repair is a tensionless technique that is performed by placing a continuous suture between the conjoined tendon and the inguinal ligament without forcibly approximating the two structures. This repair uses a very small amount of suture material for the repair compared to placing a mesh, which in turn reduces the chances of infection due to less foreign material in the repair. Darn repair is widely used in sub-Saharan Africa for both elective and emergency presentations of groin hernias [13]. There is no significant difference between Darn repair and mesh repair of inguinal hernias in terms of recurrence [14]. Our patient did develop midline abdominal wound dehiscence, possibly due to the presence of gross peritonitis and abdominal distension, but the hernia repair site healed well without any complications. There was no clinical evidence of hernia recurrence at the groin repair site at six-month follow-up.

## Conclusions

This case report highlights an unusual and rare presentation of a strangulated inguinal hernia associated with proximal ileal perforations. If there is bowel perforation in strangulated hernias, it is expected to be in the bowel segment trapped inside the inguinal canal. But in our case, the perforations were in the proximal bowel, the cause of which could not be ascertained. The edges of the perforations were not thickened or inflamed, and exploration of the entire bowel did not reveal any gross pathology. To our knowledge, this unusual presentation has not been reported previously.

A diagnostic laparoscopy, if technically feasible, is a useful tool in cases of bowel perforation and obstruction. But it should be used in very selective cases, keeping in mind the potential of iatrogenic bowel injury and a low threshold of conversion to open surgery. This case report reminds and reinforces the advice that patients with inguinal hernia, especially the indirect type, should be educated about the possibility of strangulation along with its potential complications, and get it repaired early. The standard hernia repair in adults is done by using a mesh, but it is risky in the presence of infection. The hernia repair using the nylon Darning technique is a useful alternative to prosthetic mesh repair in the presence of peritonitis.
